# A non-invasive clinical application of wave intensity analysis based on ultrahigh temporal resolution phase-contrast cardiovascular magnetic resonance

**DOI:** 10.1186/1532-429X-14-57

**Published:** 2012-08-09

**Authors:** Giovanni Biglino, Jennifer A Steeden, Catriona Baker, Silvia Schievano, Andrew M Taylor, Kim H Parker, Vivek Muthurangu

**Affiliations:** 1 Centre for Cardiovascular Imaging, UCL Institute of Cardiovascular Science, and Great Ormond Street Hospital for Children, NHS Trust, London, UK; 2 Department of Bioengineering, Imperial College London, London, UK; 3 Centre for Cardiovascular Imaging, UCL Institute of Cardiovascular Science, 30 Guildford Street, London, WC1N 1EH, UK

**Keywords:** Wave intensity analysis, Cardiovascular magnetic resonance, Hemodynamics, Spiral sequence

## Abstract

**Background:**

Wave intensity analysis, traditionally derived from pressure and velocity data, can be formulated using velocity and area. Flow-velocity and area can both be derived from high-resolution phase-contrast cardiovascular magnetic resonance (PC-CMR). In this study, very high temporal resolution PC-CMR data is processed using an integrated and semi-automatic technique to derive wave intensity.

**Methods:**

Wave intensity was derived in terms of area and velocity changes. These data were directly derived from PC-CMR using a breath-hold spiral sequence accelerated with sensitivity encoding (SENSE). Image processing was integrated in a plug-in for the DICOM viewer OsiriX, including calculations of wave speed and wave intensity. Ascending and descending aortic data from 15 healthy volunteers (30 ± 6 years) data were used to test the method for feasibility, and intra- and inter-observer variability. Ascending aortic data were also compared with results from 15 patients with coronary heart disease (61 ± 13 years) to assess the clinical usefulness of the method.

**Results:**

Rapid image acquisition (11 s breath-hold) and image processing was feasible in all volunteers. Wave speed was physiological (5.8 ± 1.3 m/s ascending aorta, 5.0 ± 0.7 m/s descending aorta) and the wave intensity pattern was consistent with traditionally formulated wave intensity. Wave speed, peak forward compression wave in early systole and peak forward expansion wave in late systole at both locations exhibited overall good intra- and inter-observer variability. Patients with coronary heart disease had higher wave speed (p <0.0001), and lower forward compression wave (p <0.0001) and forward expansion wave (p <0.0005) peaks. This difference is likely related to the older age of the patients’ cohort, indicating stiffer aortas, as well as compromised ventricular function due to their underlying condition.

**Conclusion:**

A non-invasive, semi-automated and reproducible method for performing wave intensity analysis is presented. Its application is facilitated by the use of a very high temporal resolution spiral sequence. A formulation of wave intensity based on area change has also been proposed, involving no assumptions about the cross-sectional shape of the vessel.

## Introduction

The transmission of the arterial pulse wave through the vasculature is dependent on multiple factors including: myocardial function, wall stiffness and wave reflections. Thus, analysis of wave propagation is of great clinical interest, offering insight into the integrated function of the cardiovascular system. One method by which this type of analysis can be accomplished is wave intensity analysis (WIA) [1].

In the traditional formulation of WIA, pulse wave velocity (*c*), wave reflections and energy transmission are all derived from measurements of pressure (*P*) and velocity (*U*) [2]. Pulse wave velocity can be used to assess arterial stiffness and the wave intensity patterns can be used to assess cardiac function. For instance, the intensity of the forward compression wave (FCW) is related to myocardial contractility and the forward expansion wave (FEW) is related to diastolic relaxation [3]. However, the requirement for invasive pressure measurements has prevented WIA from becoming a routine clinical test. Nevertheless, WIA does offer the possibility of more sophisticated analysis of the cardiovascular system and a non-invasive methodology is desirable.

Recently, a WIA formulation that uses diameter rather than *P* to assess wave propagation has been proposed [4]. This method is well suited to imaging techniques that assess both vessel size and flow, and was first demonstrated using ultrasound [3,5]. However, ultrasound is user dependent and limited by acoustic windows. A better imaging methodology for WIA may be phase contrast cardiovascular magnetic resonance (PC-CMR).

PC-CMR is a reference standard method of measuring blood velocity and has been successfully used to assess vessel distension [6,7]. However, the calculation of *c* in WIA requires very high temporal resolution velocity and distension data. In addition, accurate arterial distension data can only be acquired with high spatial resolution PC-CMR. Unfortunately, high spatio-temporal resolution PC-CMR has a long acquisition time and cannot be performed in a breath hold. Thus, in previous studies free breathing PC-CMR with multiple signal averages has been used [8]. Although this approach does provide the necessary resolution, it suffers from poor vessel wall delineation due to respiratory artefacts. An alternative approach is to use highly accelerated PC-CMR sequences. Such sequences have the capability of acquiring high-resolution data in a short breath hold. As such data are not corrupted by respiratory artefact, and vessel size should be more accurately measured. In this study, a previously validated spiral PC-CMR sequence accelerated with sensitivity encoding (SENSE) [9] was used to acquire high spatio-temporal resolution aortic flow data within a breath-hold. The PC-CMR data were then analysed in a semi-automated fashion deriving *c*, and wave intensity information.

The aims of this study were: i) to demonstrate the feasibility of performing WIA in the ascending and descending aorta of normal volunteers using spiral PC-CMR; ii) to assess intra- and inter-observer variability of the WIA processing package; iii) to use WIA to capture differences between normal volunteers and patients with coronary artery disease, thus showing clinical relevance of the integrated methodology.

## Methods

### Non-invasive wave intensity analysis

A previous study using MR to perform WIA has used vessel diameter as a surrogate of pressure [8]. However, vessel area is a more direct measure when using PC-CMR. Net wave intensity can be derived directly from measurements of *U* and *A*, but the separation of wave intensity into its forward and backward components requires knowledge of the wave speed *c*. The wave speed is related to the arterial distensibility through the Bramwell-Hill equation:

(1) $$c^{2}=\frac{1}{\rho D}=\frac{AdP}{\rho dA}$$

where *ρ* is the density of blood, and *D* is the vessel distensibility, which equals the relative change in area (*dA/A*) divided by the change in pressure (*dP*).

Wave speed is also related to *dP* and the change in velocity (*dU*) through the waterhammer equation: [10]

(2) $$dP_{\pm }=\pm \rho cdU_{\pm }$$

where + refers to forward waves and – to backward-travelling waves, i.e. “away from the heart” and “toward the heart”. Therefore we can replace *dP* in equation 1 with equation 2 and formulate *c* using only non-invasive parameters.

(3) $$\text{c}=\frac{AdU}{dA}=\frac{dU}{dlnA}$$

From equation 3, it can be seen that *c* is the gradient of the *U-lnA* loop in early systole, when no reflected waves are expected. Thus, *c* can be calculated in a similar way as per the gradient of the *P-U* loop [11], a method often used in traditional WIA.

Equation 3 can also be re-arranged to produce the water hammer equation in terms of U and A

(4) $$dU_{\pm }=\pm cdlnA_{\pm }$$

In WIA waves are regarded as a summation of incremental wave fronts [2], and it is thus possible to separate the *U* and *A* curves into the respective forward and backward components:

(5) $$dU=dU_{+}+dU_{-}$$

(6) $$dlnA=dlnA_{+}+dlnA_{-}$$

Then the separated curves can be calculated by substitution of equation 4 into equations 5 and 6:

(7) $$dU_{\pm }=\frac{1}{2}\left(dU\pm cdlnA\right)$$

(8) $$dlnA_{\pm }=\frac{1}{2}\left(dlnA\pm \frac{1}{c}dU\right)$$

The separated U and A curves can then be calculated by summing the separated area and velocity derivatives. Finally, the separated wave intensity *dI*_*A*_ (where subscript A denotes the area formulation) can be calculated as the product of *dU*_*±*_ and *dlnA*_*±*_, whereas net *dI*_*A*_ is defined as the product of *U* and *lnA* differentials:

(9) $$dI_{A}=dUdlnA$$

A full derivation of the area formulation of wave intensity from first principles is provided in Additional file 1.

### Study population

The study population consisted of 15 healthy adults (11 male, 30 ± 6 years of age) and 15 significantly (p < 0.0001) older patients with confirmed coronary artery disease referred for clinical CMR (10 male, 61 ± 13 years of age). Exclusion criteria were: i) MR incompatible implants, ii) claustrophobia, iii) pregnancy and iv) any cardiovascular disease within the volunteers cohort. The local research ethics committee approved the study.

### MR protocol

All imaging was performed on a 1.5 T MR scanner (Avanto, Siemens Medical Solutions, Erlangen, Germany) using two spine coils and one body-matrix coil (giving a total of 12 coil elements). Phase-contrast MR was performed using a previously validated prospectively triggered, velocity encoded spiral spoiled gradient echo sequence (TE/TR: 1.9/4.8 ms, FOV: 400×400×6 mm, matrix: 192×192, VENC: 180 cm/s) [9]. A uniform-density spiral trajectory was used, with 60 spiral interleaves, undersampled by a factor of four (so only 15 spiral interleaves were acquired for each cardiac phase). One spiral interleave was acquired per R-R interval, meaning that 15 R-R intervals were used to acquire all of the data. One additional R-R interval was required at the beginning of imaging to reach a steady state. Thus, the average breath-hold time was 11 s. The sampling pattern was rotated for each cardiac phase so that four consecutive cardiac phases comprised a fully sampled k-space with 60 spiral readouts. The resultant temporal resolution was 9.6 ms and the spatial resolution was 2.1×2.1 mm. In all subjects, PC-CMR was performed in the ascending aorta at the level of the bifurcation of the pulmonary arteries. In addition, in volunteers PC-CMR was also performed in the descending aorta at the level of the diaphragm.

### Image processing

All images were processed using an in-house written plug-in for the open source DICOM software OsiriX (OsiriX Foundation, Geneva, Switzerland) [12].Segmentation of the aorta was performed on the modulus image using a previously validated semi-automatic registration-based algorithm [13].In order to ensure accurate delineation of the vessel wall, the ability to alter regions of interest (ROI) was available to the operator. The final ROI was used to both calculate the aortic cross-sectional area (*A*) and prescribe the area in the phase image from which the mean aortic velocity (*U*) was calculated. While the propagation algorithm was previously validated [13], the possibility of performing minor adjustments to the ROI was provided to ensure the goodness of the area curve. The resultant *U* and *A* curves were interpolated to a 1 ms temporal resolution and the *A* curve was smoothed using a Savitzky-Golay filter [14] of 3^rd^ order with 100 points window size. Then *U* and *lnA* were combined into a loop whose optimal linear portion was automatically selected (by finding the portion of the loop in early systole with the best linear fit) and *c* was calculated from its gradient. Combining knowledge of c with *dU* and *dlnA* data, the net, forward and backward *dI*_*A*_ were finally plotted. These wave intensity data were post-processed in MATLAB (Mathworks Inc, Natick, MA, USA), where peaks of the FCW and FEW were measured.

### Statistics

Values are presented as mean ± standard deviation. Intra-observer variability was verified by the same operator repeating the analysis one week apart. Inter-observer variability was assessed by two different blinded operators performing the analysis separately. Values of *c*, peak FCW and peak FEW from the volunteers cohort were tested for intra- and inter-observer variability by quantifying the intraclass correlation coefficient (ICC) (two way random effects model, SPSS, IBM), both in the ascending and descending aorta. Bias and limits of agreement from Bland-Altman analysis were also assessed. Differences between the two cohorts (volunteers *vs.* patients) were assessed using unpaired *t* test, and p < 0.05 was regarded as statistically significant.

## Results

### Feasibility

**Figure 1:**
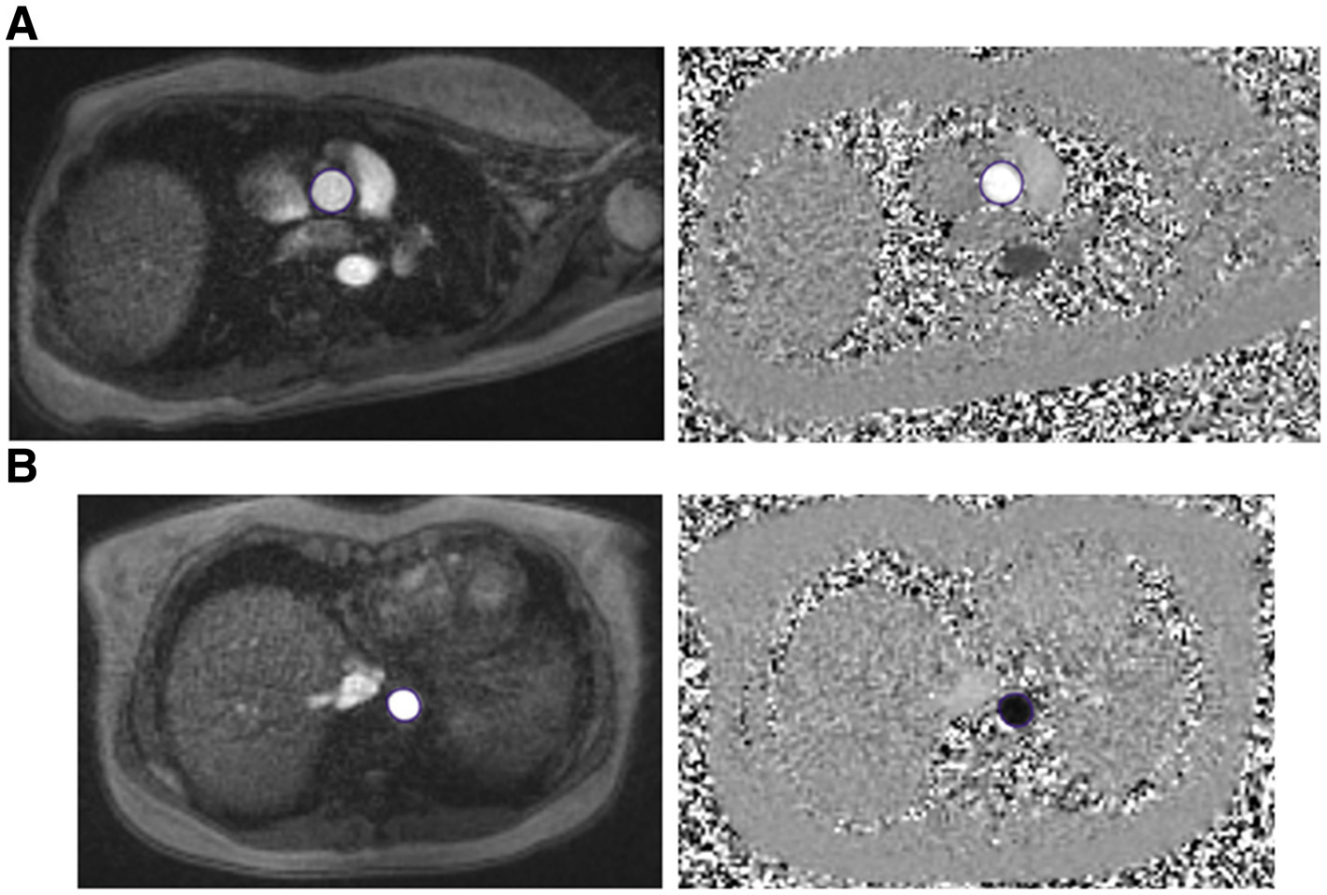
**Phase-contrast CMR data.** Sample of modulus and phase images from the ascending (**A**) and descending (**B**) aorta of a volunteer at peak systole.

**Figure 2:**
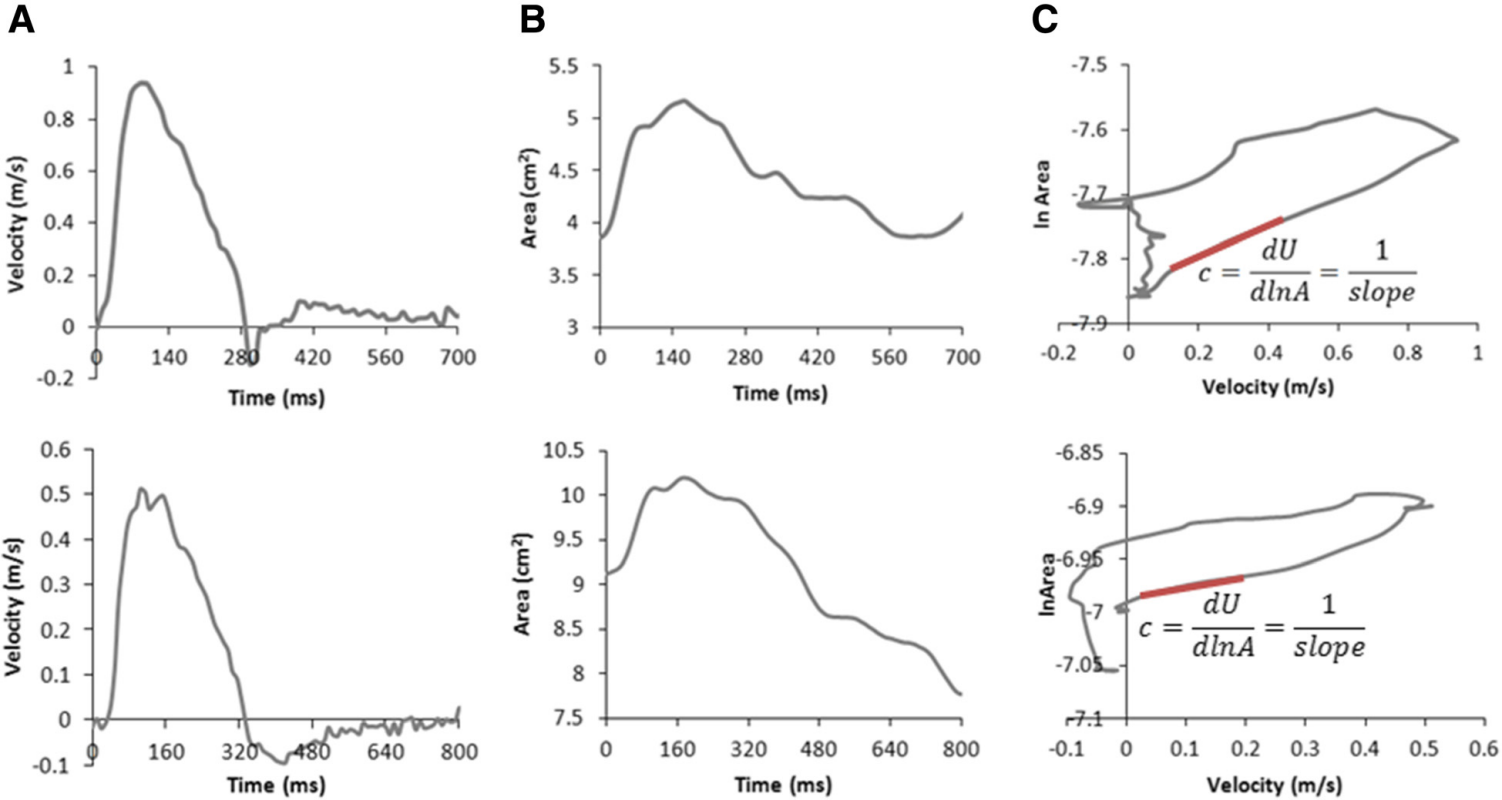
**Calculation of wave speed.** Samples of velocity (**A**) and area (**B**) curves calculated with the plug-in. These data are combined in a loop (**C**) whose linear slope in early systole (highlighted in red) yields wave speed *c*. Waveforms from both a volunteer (*top row*) and a patient (*bottom row*) are shown.

**Figure 3:**
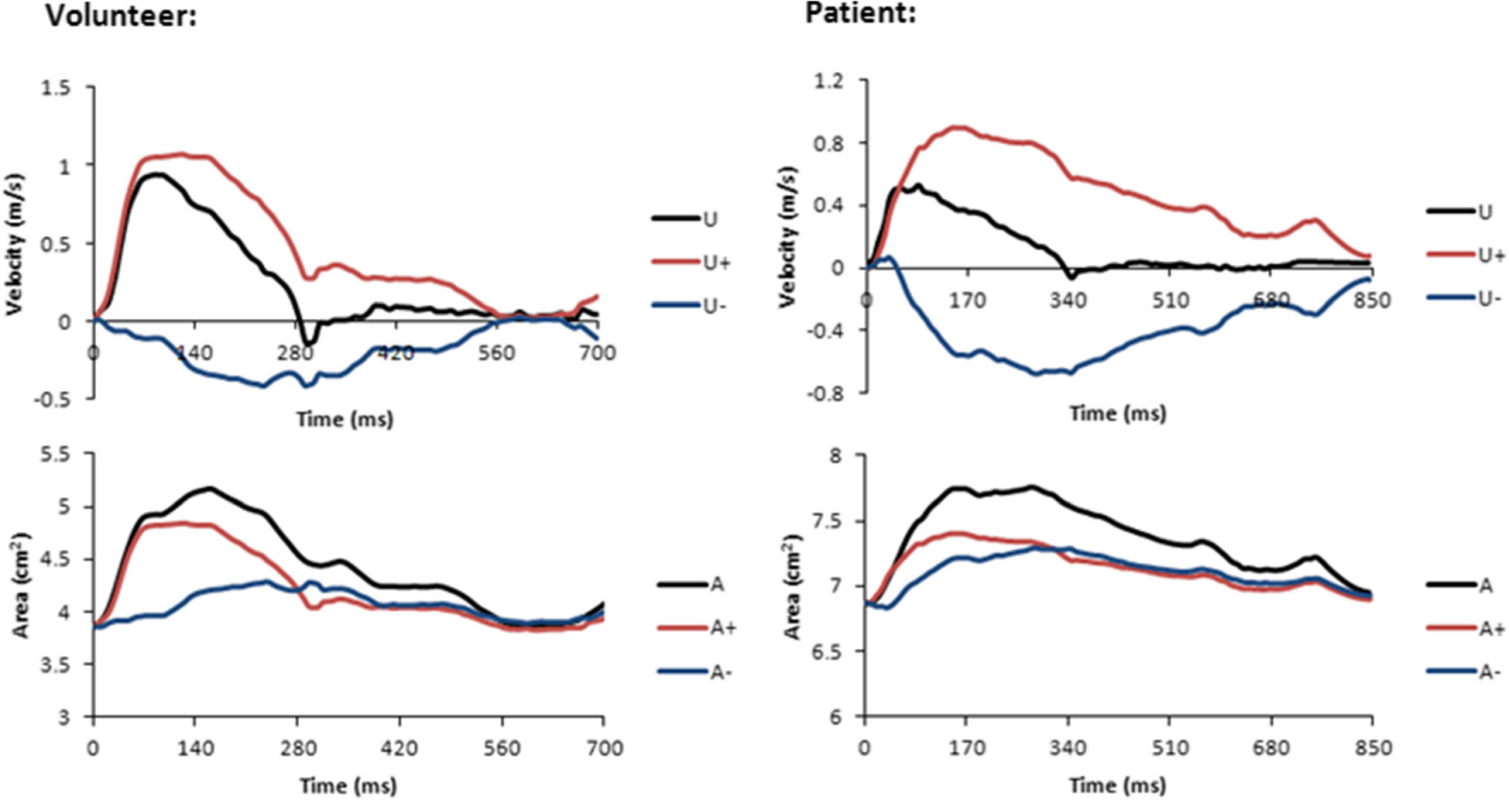
**Waveform separation.** Measured velocity *U* and area *A* can be separated into forward (+) and backward (−) components. Data from one of the volunteers and one of the patients are presented.

**Figure 4:**
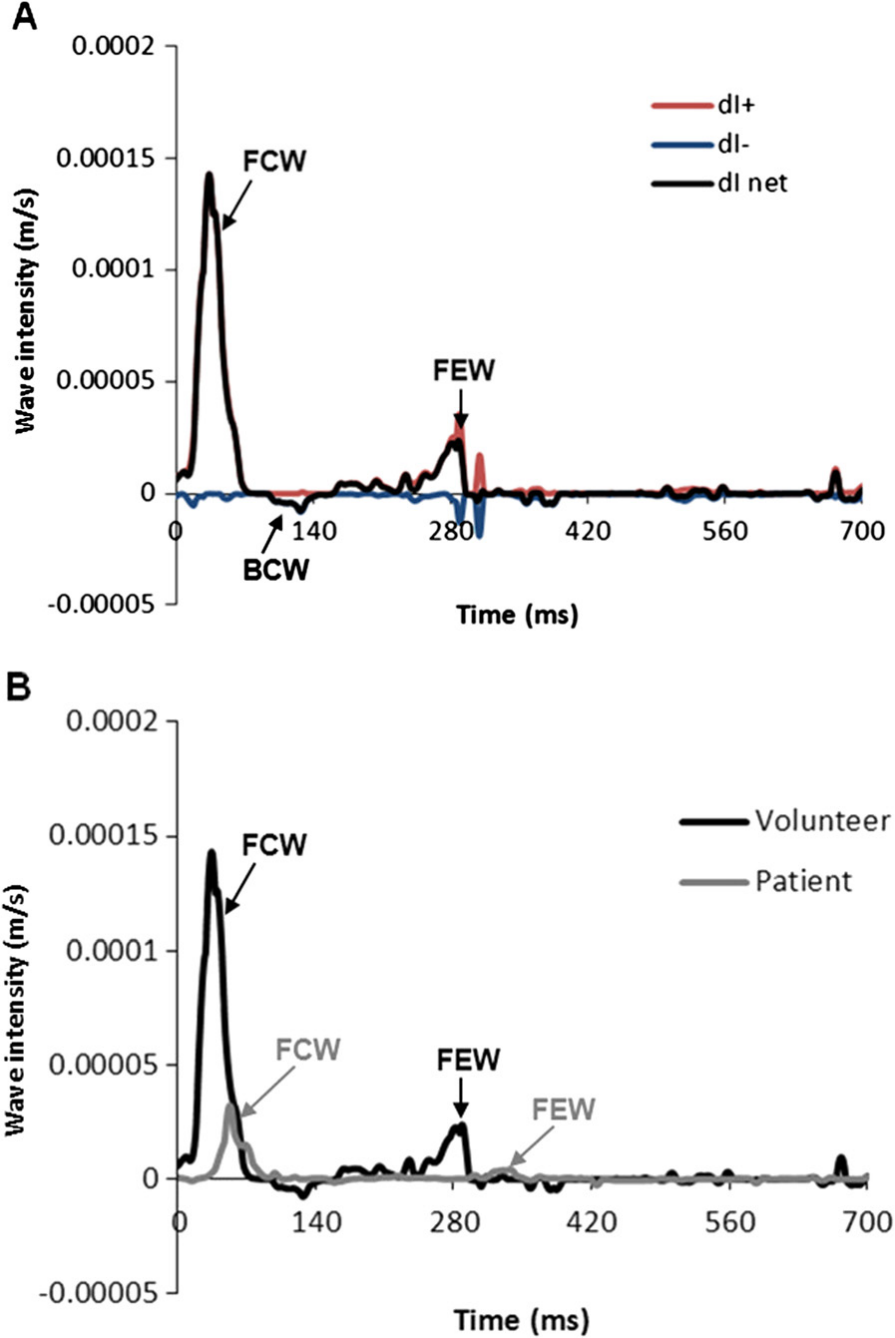
**Wave intensity pattern and net wave intensity comparison between patients and volunteers.** Pattern of separated and net wave intensity (*dI*) of one volunteer (A), highlighting a typical pattern with: dominant forward compression wave in early systole (FCW), followed by a backward compression wave (BCW) and a forward expansion wave (FEW) in late systole. Comparison with a net *dI* pattern of a patient with coronary heart disease (B) shows a clear difference in FCW and FEW peaks magnitude.

Phase contrast MR data were successfully acquired in all volunteers and patients within a breath hold (Figure 1). All data were successfully analysed using the in-house software, taking approximately 15 minutes per case. Figure 2 shows the *U* and *A* curves for a volunteer, as well as the *U-lnA* loop used for calculation of *c*. In volunteers, the values of *c* were within the accepted normal range (5.8 ± 1.3 m/s in the ascending aorta and 5.0 ± 0.7 m/s in the descending aorta). An example of separated *U* and *A* curves is shown in Figure 3. These curves are similar to literature examples of invasive *P* and *U* curves, and non-invasive *D* and *U* curves [15].The wave intensity pattern was also in keeping with previous data, with a dominant forward compression wave (FCW) followed by a small backward compression wave and a forward-traveling expansion wave at the end of systole (Figure 4).

### Reproducibility

The ICC’s for intra-observer and inter-observer variability are reported in Table 1. In the ascending aorta *c* and FCW were both highly reproducible. However, FEWin the ascending aorta had poor reproducibility. In the descending aorta *c*, FCW and FEW all had reasonable reproducibility (although reproducibility of FCW and *c* was slightly lower than in the ascending aorta).

**Table 1:** Assessment of intra- and inter-observer variability of ascending and descending aortic measurements

**INTRA-OBSERVER**						
**Location**	**Ascending aorta (n = 15)**			**Descending aorta (n = 15)**		
**Variable**	***c (m/s)***	***peak FCW (×10***^***-5***^ ***m/s)***	***peak FEW (×10***^***-5***^ ***m/s)***	***c (m/s)***	***peak FCW (×10***^***-5***^ ***m/s)***	***peak FEW (×10***^***-5***^ ***m/s)***
**ICC**	0.961	0.894	0.391	0.976	0.869	0.88
**95 % CI**	0.887-0.987	0.715-0.963	−0.153-0.754	0.929-0.992	0.654-0.984	0.680-0.958
**Bias**	0.03	−0.2	−0.1	−0.1	0.3	0.2
**Limits of agreement**	−0.7-0.76	−5.9-5.5	−2.9-2.6	−0.4-0.2	−6.6-7.2	−1.2-1.6
**INTER-OBSERVER**						
**Location**	**Ascending aorta (n = 15)**			**Descending aorta (n = 15)**		
**Variable**	***c (m/s)***	***peak FCW (×10***^***-5***^ ***m/s)***	***peak FEW (×10***^***-5***^ ***m/s)***	***c (m/s)***	***peak FCW (×10***^***-5***^ ***m/s)***	***peak FEW (×10***^***-5***^ ***m/s)***
**ICC**	0.937	0.84	0.315	0.635	0.694	0.863
**95 % CI**	0.903-0.989	0.588-0.943	−0.238-0.713	0.201-0.860	0.301-0.886	0.641-0.952
**Bias**	−0.07	3.8	0.02	−0.2	4.2	0.3
**Limits of agreement**	−0.96-0.83	−2.1-9.6	−1.8-1.9	−1.5-1.1	−5.1-13.5	−1.2-1.9

*ICC* = intraclass correlation coefficient; *CI* = confidence interval; *c* = wave speed, *FCW* = forward compression wave; *FEW* = forward expansion wave.

### Comparison between normal volunteers and patients

**Figure 5:**
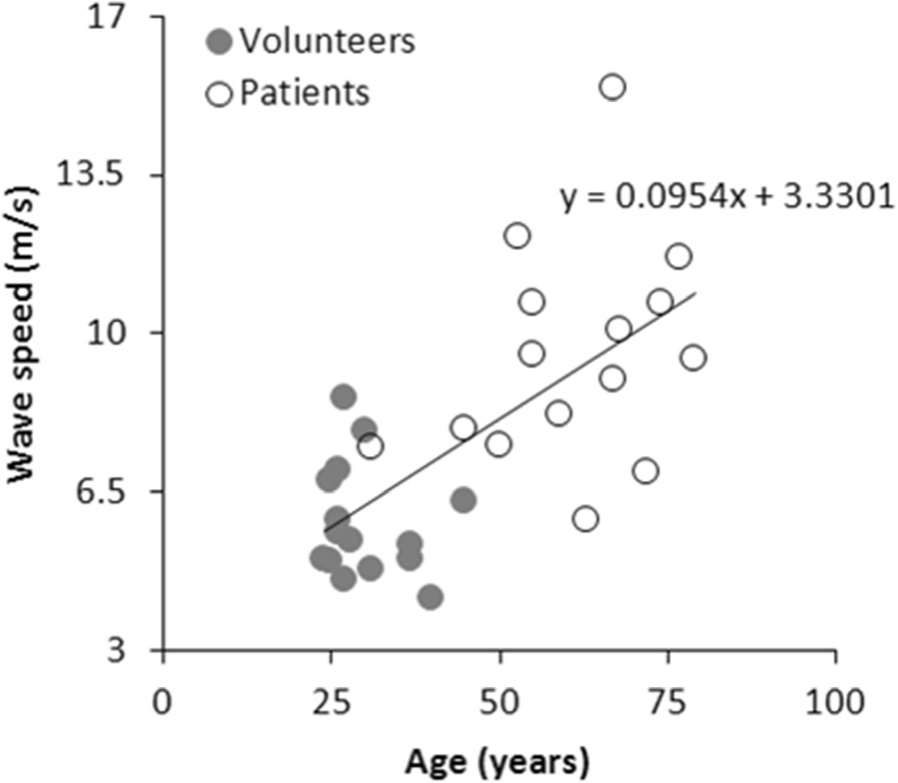
**Relationship between wave speed and age.** Physiological relationship between increasing age and increasing wave speed, obtained pooling data from the two cohorts of volunteers (*full dots*) and patients (*empty dots*).

Figure 4 shows an example of the wave intensity plot for a patient in which the FCW and FEW are obviously lower than in volunteers. In fact, there were large significant (p < 0.05) differences in *c*, FCW and FEW between volunteers and older patients with coronary heart disease (Table 2). Such differences were most likely age-related, as demonstrated by the relationship between increasing age and increasing *c* (Figure 5).

**Table 2:** Comparison of wave speed and wave intensity peaks between healthy volunteers and patients

**Variable**	**Volunteers*(n = 15)***	**Patients*(n = 15)***	**p value**
c (m/s)	5.8 ± 1.3	9.5 ± 2.4	<0.0001
peak FCW (×10^-5^ m/s)	11.5 ± 5.2	3.1 ± 2.5	<0.0001
peak FEW (×10^-5^ m/s)	1.6 ± 0.7	0.6 ± 0.4	<0.0005

*c* = wave speed; *FCW* = forward compression wave; *FEW* = forward expansion wave.

## Discussion

In this study we have shown that it possible to reliably perform WIA using PC-CMR. This technique is dependent on a rapid high resolution PC-CMR sequence that was acquired in a breath hold. Thus, images were not degraded by respiratory artefact, which improved the assessment of vessel distension. We have further demonstrated that WIA measures can easily and robustly differentiate normal volunteers from older patients with coronary artery disease, detecting age-related differences in arterial wall stiffness and indicating a potential for differentiating between health and disease. This suggests that the technique does have clinical utility, although it will be important to test in other patient groups with milder disease.

Wave intensity analysis is a proven technique that allows comprehensive assessment of cardiac and vascular function. Previous invasive studies have shown that the FCW correlates with left ventricular dP/dt (myocardial contractility), while FEW correlates with left ventricular Tau (diastolic relaxation) [3]. In addition, through the measurement of *c*, arterial wall stiffness can also be evaluated*.* Unfortunately, the traditional formulation of WIA requires invasive pressure measurements and this has limited its uptake into routine clinical practice.

Recently, a formulation of WIA has been proposed that utilizes vessel distension rather pressure. Initial phantom experiments using ultrasound to measure velocity and distension have shown excellent agreement with the traditional invasive methodology [16]. However, ultrasound does have some disadvantages such as operator dependence and limited acoustic windows. An alternative to ultrasound is PC-CMR, which has the ability to simultaneously measure velocity and distension in the true short axis of any vessel. Unfortunately, the requirement for high spatio-temporal resolution data leads to long acquisition times and previous studies have had to use free breathing PC-CMR. This is problematic, as respiratory artefact can lead to significant edge blurring and inaccurate vessel distension measurements. In this study, we combined efficient spiral *k*-space trajectories with SENSE to acquire high resolution PC-CMR data in a short breath hold. This sequence has previously been validated and offers a way of acquiring velocity and distension data without respiratory contamination of the images. However, this sequence does suffer from reduced SNR due to undersampling, and edge blurring due to spiral imaging. Because of the need of making the sequence clinically useful (i.e. acquired in a short, manageable breath hold), time scan was pushed as fast as possible, requiring a SENSE factor of 4. This solution admittedly impinges on SNR, but nevertheless image quality was sufficient to semi-automatically segment the data in all cases. The other difference between this study and a previous retrospective MR study [8] was the use of area rather than diameter for evaluation of vessel distension. Diameter is the only measurement available in combined ultrasound imaging and Doppler assessment. However in MR, the vessel short axis is acquired and this lends itself to assessment of area. Area measurement has the benefits of not assuming circularity, as well as providing better reproducibility. Thus, it can be argued that area is the most valid way of measuring vessel distension using MR. Of course, this approach does require vessel segmentation of every frame, which is not clinically feasible when done manually. Thus, we used a semi-automatic segmentation algorithm that allows rapid processing with minimal user manipulation of the ROI’s. By combining rapid MR imaging with rapid processing we believe that this implementation of WIA can be used successfully in the clinical environment.

It should be noted that in this study a time correction [17] was not applied to the data as the sampling frequency was the same in all cases. However, if the sampling frequency were different in different patient groups (e.g. to reduce breath hold time) then time correction would be necessary. This would be best done by using dU/dt x dlnA/dt in the WIA formulation and could be easily implemented in our plug-in.

In volunteers, WIA patterns and values of *c* produced by this technique were in agreement with previous invasive and non-invasive studies [18,19].Values of c also increased with age as observed in previous studies [20,21], although combining healthy subjects and patients may have confounded this result. Furthermore, there were large differences in *c*, FCW and FEW between older patients with coronary artery disease and younger healthy volunteers. This difference is perhaps unsurprising, due to the significantly (p < 0.0001) older age of the patients’ cohort, reflecting stiffer vessels, as well as their likely reduced systolic and diastolic function. Nevertheless, it does demonstrate the clinical utility of non-invasive WIA. In addition, these measures are more load independent than other common MR measures (such as ejection fraction) and therefore do provide added value [22].

For a new technique to be accepted clinically it must have proven reproducibility. We have shown that in normal volunteers there is good reproducibility for *c* and FCW in both the ascending and descending aorta. However, in the ascending aorta there was poor reproducibility for FEW compared to the descending aorta. This is probably because of the low-end systolic signal in the ascending compared to descending aorta, due to reduced in-flow enhancement. This hampers automated segmentation and means that currently more user interaction is required, introducing greater errors in measurement. Possible solutions to this problem may lay in the development of new steady state free precession PC-CMR sequences that have high blood signal throughout the cardiac cycle. Such sequences should provide images that are much more amenable to automated segmentation with very little user interaction. Nevertheless, it should be noted that there is an almost 3-fold difference in FEW between patients and volunteers. Thus, even with poor reproducibility it is still possible to differentiate between health and disease. Of course, greater reproducibility is necessary if milder forms of disease are to be diagnosed using this technique.

In terms of the detection of the linear part of the loop for calculation of wave speed, the goodness of the linear fit was demonstrated by the correlation coefficients, reported in Table 3 for ascending and descending aortic data from the volunteers’ cohort. This is achieved without the need for shifting one of the two signals (i.e. either A and U) for time-alignment, as instead often necessary with P and U data, thus representing an additional benefit of using CMR-derived data. It was also shown that interpolating the data and the type of interpolation used (linear *vs.* PCHIP) did not affect the quality of the A and U waveforms, nor the wave intensity pattern.

**Table 3:** **R**^**2**^**coefficients for the linear part of the lnA-U loops in the volunteers’ cohort, both for ascending and descending aortic data, indicating the goodness of the linear fit**

**Volunteer #**	**R**^**2**^**coefficient linear part**	
	**Ascending aorta**	**Descending aorta**
1	0.9896	0.9804
2	0.9998	0.9952
3	0.997	0.995
4	0.9842	0.991
5	0.9962	0.9983
6	0.9935	0.9923
7	0.9926	0.9999
8	0.9942	0.9935
9	0.9995	0.9962
10	0.9997	0.9954
11	0.9938	0.9986
12	0.9924	0.9934
13	0.9892	0.9971
14	0.9861	0.9971
15	0.9885	0.9971
***Average***	***0.993***	***0.995***

### Limitations

The main limitation of this technique is that the spiral SENSE sequence is prospectively gated and thus does not acquire data in end diastole. For WIA in central vessels this is not a major problem as there are few reflected waves in this part of the cardiac cycle. However, for other parts of the vasculature inability to acquire the whole cardiac cycle may limit this technique. Future work on retrospective gating is thus important in these situations.

## Conclusion

A non-invasive method for calculating wave intensity based on PC-CMR data has been presented. This method depends on rapid breath-hold PC-CMR and semi-automated image processing. We have shown that this technique is reproducible for *c* and FCW and it is able to differentiate between patients and volunteers of different age and underlying cardiovascular condition. In the future this methodology may be useful in detection of more subtle changes in vascular and cardiac function in diseases like systemic and pulmonary hypertension.

## Competing interests

The authors declare that they have no competing interests.

## Authors’ contributions

GB was involved in designing the study, compiling and testing the plug-in, collecting and analysing data, performing statistical analysis; JAS was involved in compiling the plug-in and developing the MR sequence; CB was involved in the design of study and contributed to the interpretation of data; SS and AMT have been involved in designing the study and revising the manuscript for important intellectual content; KHP was involved in wave intensity analysis formulation and data interpretation; VM supervised the acquisition of data and implementation of the MR sequence, and was involved in drafting and revising the manuscript. All authors read and approved the final manuscript.

## Supplementary Material

Additional file 1 Area and velocity formulation of wave intensity. Click here for file
