# Supplementary material for: A non-invasive clinical application of wave intensity analysis based on ultrahigh temporal resolution phase-contrast cardiovascular magnetic resonance
Source: J Cardiovasc Magn Reson. 2012 Aug 9;14(1):57. doi: 10.1186/1532-429X-14-57 (PMC3472227; doi:10.1186/1532-429X-14-57)
Supplement: Additional file 1 — Area and velocity formulation of wave intensity. [file 1532-429X-14-57-S1.pdf]

Appendix 1 – Area and velocity formulation of wave intensity

In this study we propose that it is useful to express wave intensity in terms of A and U when working with MR measurements and thus the relevant derivations from first principles follow.

In 1-D analysis, U, A and P are taken as functions only of axial length (x) and time (t). Defining U as mean velocity over a cross-section and hence UA as the flow rate, the equations of conservation of mass and conservation of momentum are:

$$\frac{\partial A}{\partial t}+\frac{\partial (UA)}{\partial x}=0 \qquad \textbf{(1)}$$

$$\frac{\partial U}{\partial t}+U\frac{\partial U}{\partial x}=-\frac{1}{\rho}\frac{\partial P}{\partial x}-FU \qquad \textbf{(2)}$$

where ρ is the fluid density and F is the resistance arising from the viscosity of the flowing medium. A tube law relating the area of the vessel and the pressure is then assumed:

$$A=A(P) \qquad \textbf{(3)}$$

and the gradient of P can be expressed in terms of A:

$$\frac{dP}{dx}=\frac{\left(\frac{dA}{dx}\right)}{\left(\frac{dA}{dP}\right)} \qquad \textbf{(4)}$$

The resulting conservation equations in matrix form are:

$$\begin{pmatrix}A\\U\end{pmatrix}_t+\begin{pmatrix}U & A\\ \frac{1}{\rho\frac{dA}{dP}} & U\end{pmatrix}\begin{pmatrix}A\\U\end{pmatrix}_x=\begin{pmatrix}0\\FU\end{pmatrix} \qquad \textbf{(5)}$$

Where we use subscript notation for partial derivatives. The eigenvalues of the matrix multiplying the x-derivatives, according to the method of characteristics, are:

$$\lambda_{\pm}=U\pm c \qquad \textbf{(6)}$$

where we have defined the wave speed, c:

$$c(x,t)=\sqrt{\frac{A}{\rho\frac{dA}{dP}}}=\sqrt{\frac{1}{\rho D}} \qquad \textbf{(7)}$$

where D is vessel distensibility, as firstly derived by Bramwell and Hill.

Following the method of characteristics, along the characteristic directions dx/dt = U ± c, the conservation equations (1 and 2) become:

$$\frac{dA}{dt} - (U \pm c)\frac{\partial A}{\partial x} + U\frac{\partial A}{\partial x} + A\frac{\partial U}{\partial x} = 0 \qquad \textbf{(8)}$$

$$\frac{dU}{dt} - (U \pm c)\frac{\partial U}{\partial x} + \frac{c^2}{A}\frac{\partial A}{\partial x} + U\frac{\partial U}{\partial x} = -FU \qquad \textbf{(9)}$$

By multiplying equation (8) by a factor of $\left(\pm\frac{c}{A}\right)$ and then substituting it into equation (9) for $\frac{\partial A}{\partial x}$, it results that:

$$\frac{dU}{dt} \pm c\frac{d\ln A}{dt} = -FU \qquad \textbf{(10)}$$

given dA/A = dlnA. Note that + is valid along the forward characteristic (dx/dt = U + c) and – along the backward characteristic (dx/dt = U – c).

The changes in velocity (dU) and the fractional changes in area (dlnA) are related by the waterhammer equation:

$$dU_{\pm} = \pm cd\ln A_{\pm} \qquad \textbf{(11)}$$

Assuming that the forward and backward waves are additive, the measured dU and dlnA can be separated into their forward (+) and backward (–) components by using the waterhammer equations:

$$dU_{\pm} = \frac{1}{2}\left(dU \pm cd\ln A\right) \qquad \textbf{(12)}$$

$$d\ln A_{\pm} = \frac{1}{2}\left(d\ln A \pm \frac{1}{c}dU\right) \qquad \textbf{(13)}$$

Finally, wave intensity can be defined in terms of area (dI_A), as:

$$dI_A = dUd\ln A \qquad \textbf{(14)}$$

It can be shown that the net wave intensity can be divided into the forward and backward intensities:

$$dI_A = dI_{A(+)} + dI_{A(-)} \qquad \textbf{(15)}$$

with the separated dI_A expressed as:

$$dI_{A(\pm)} = \pm\frac{c}{4}\left(d\ln A \pm \frac{1}{c}dU\right)^2 \qquad \textbf{(16)}$$
